# Pharmacological Treatments and Therapeutic Targets in Muscle Dystrophies Generated by Alterations in Dystrophin-Associated Proteins

**DOI:** 10.3390/medicina60071060

**Published:** 2024-06-27

**Authors:** Alexandra Luna-Angulo, Carlos Landa-Solís, Rosa Elena Escobar-Cedillo, Francisco Javier Estrada-Mena, Laura Sánchez-Chapul, Benjamín Gómez-Díaz, Paul Carrillo-Mora, Hamlet Avilés-Arnaut, Livier Jiménez-Hernández, Dulce Adeí Jiménez-Hernández, Antonio Miranda-Duarte

**Affiliations:** 1 División de Neurociencias Clinicas, Instituto Nacional de Rehabilitación “Luis Guillermo Ibarra Ibarra”, Calzada México-Xochimilco, No. 289, Arenal de Guadalupe, Tlalpan, Ciudad de México 14389, Mexico; 2 Unidad de Ingeniería de Tejidos, Terapia Celular y Medicina Regenerativa, División de Biotecnología, Instituto Nacional de Rehabilitación “Luis Guillermo Ibarra Ibarra”, Calzada México-Xochimilco, No. 289, Arenal de Guadalupe, Tlalpan, Ciudad de México 14389, Mexico; 3 Departamento de Electromiografía y Distrofia Muscular, Instituto Nacional de Rehabilitación “Luis Guillermo Ibarra Ibarra”, Calzada México-Xochimilco, No. 289, Arenal de Guadalupe, Tlalpan, Ciudad de México 14389, Mexico; 4 Laboratorio de Biología Molecular, Universidad Panamericana, Facultad de Ciencias de la Salud, Augusto Rodin 498, Ciudad de México 03920, Mexico; 5 Departamento de Medicina Genómica, Instituto Nacional de Rehabilitación “Luis Guillermo Ibarra Ibarra”, Calzada México-Xochimilco, No. 289, Arenal de Guadalupe, Tlalpan, Ciudad de México 14389, Mexico; 6 Facultad de Ciencias Biológicas de la Universidad Autónoma de Nuevo Leon, Av. Universidad s/n Ciudad Universitaria, San Nicolas de los Garza 66455, Mexico; 7 LJ Comunicación Gráfica, Agua Marina #2827, Residencial Victoria, Guadalajara 44986, Mexico

**Keywords:** muscular dystrophy, fibrosis, drug repositioning, dystrophin-associated proteins

## Abstract

Muscular dystrophies (MDs) are a heterogeneous group of diseases of genetic origin characterized by progressive skeletal muscle degeneration and weakness. There are several types of MDs, varying in terms of age of onset, severity, and pattern of the affected muscles. However, all of them worsen over time, and many patients will eventually lose their ability to walk. In addition to skeletal muscle effects, patients with MDs may present cardiac and respiratory disorders, generating complications that could lead to death. Interdisciplinary management is required to improve the surveillance and quality of life of patients with an MD. At present, pharmacological therapy is only available for Duchene muscular dystrophy (DMD)—the most common type of MD—and is mainly based on the use of corticosteroids. Other MDs caused by alterations in dystrophin-associated proteins (DAPs) are less frequent but represent an important group within these diseases. Pharmacological alternatives with clinical potential in patients with MDs and other proteins associated with dystrophin have been scarcely explored. This review focuses on drugs and molecules that have shown beneficial effects, mainly in experimental models involving alterations in DAPs. The mechanisms associated with the effects leading to promising results regarding the recovery or maintenance of muscle strength and reduction in fibrosis in the less-common MDs (i.e., with respect to DMD) are explored, and other therapeutic targets that could contribute to maintaining the homeostasis of muscle fibers, involving different pathways, such as calcium regulation, hypertrophy, and maintenance of satellite cell function, are also examined. It is possible that some of the drugs explored here could be used to affordably improve the muscular function of patients until a definitive treatment for MDs is developed.

## 1. Introduction

Muscular dystrophies (MDs) are a clinically heterogeneous group of inherited diseases characterized by progressive skeletal muscle degeneration and weakness with variable extra-muscular manifestations. There are several types of MDs, which vary in terms of age of onset, severity, and pattern of the affected muscles. All types of MDs worsen over time, and many patients will eventually lose their ability to walk. In addition to skeletal muscle, the heart could be affected in some types of MDs, resulting in cardiac complications, such as dilated cardiomyopathy and arrhythmias. Respiratory failure is also common due to weakness of the breathing muscles, which might limit the lifespan of affected individuals. The gastrointestinal system, spine, eyes, brain, and other organs can also be affected [1,2]. Quality of life is severely impaired in most patients suffering from an MD; therefore, interdisciplinary management is required to offer an opportune and adequate therapeutic strategy (e.g., rehabilitation, cardiac, respiratory, and orthopedic management) in order to improve the quality and duration of life.

The classification of MDs was originally based on the pattern of weakness, mode of inheritance, and age of onset. Nevertheless, advances in molecular genetics have transformed their classification and, at present, there are 61 types of MDs (without counting congenital dystrophies). According to a recent meta-analysis, the global prevalence of MDs is 3.6 per 100,000 people. Duchenne muscular dystrophy (DMD) and the less severe form, Becker muscular dystrophy (BMD), are dystrophinopathies with an X-linked inheritance. They are the most common form of MDs, and their prevalence has been estimated at 4.8 and 1.6 per 100,000 people, respectively [3]. Alterations in the dystrophin-associated proteins (DAPs) may result in some form of limb girdle muscular dystrophy (LGMD), which includes more than 30 autosomally inherited disorders, although autosomal recessive is the most common form of inheritance. The overall prevalence of LGMD worldwide has been estimated to be 1 in 14,500–45,000; however, there is a wide variation in the prevalence of LGMD subtypes in different ethnicities [4]. In Mexico, the estimated prevalence found in a cohort of MDs was 52.36% for dystrophinopathies, 18.40% dysferlinopathies, 14.15% sarcoglycanopathies, 11.32% calpainopathies, 1.89% merosinopathies, 1.42% caveolinopathies, and 0.47% emerinopathies [5]. In this study, we focus on the MDs related to dystrophin-associated protein (DAP) complex [6,7,8].

**Figure 1:**
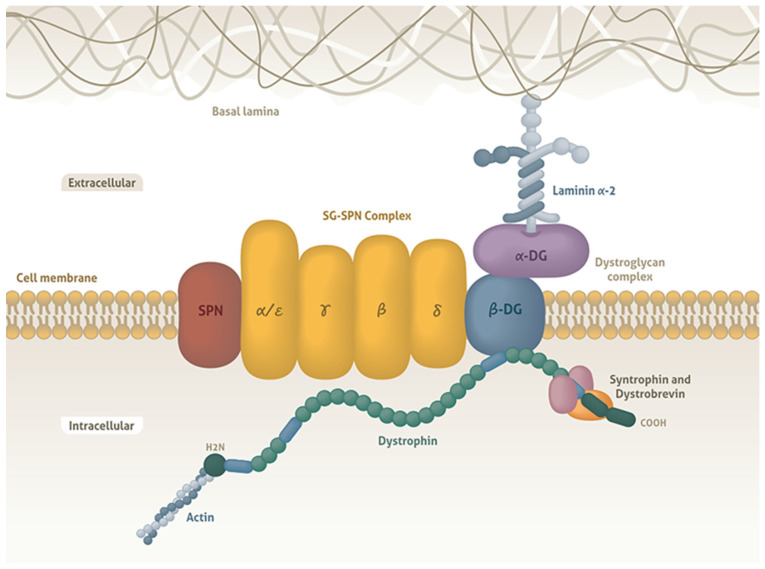
Scheme of the dystrophin-associated protein complex (DAP). In the intracellular region of the muscle fiber, dystrophin binds to actin by its amino-terminal domain (H2N) and to the membrane through the dystroglycan complex (α-, β-DG) by its carboxy-terminal domain (COOH). In turn, α-, β-DG binds to the sarcoglycan–sarcospan complex (SG-SPN), which comprises transmembrane proteins, and to the basal lamina through the extracellular protein laminin α-2. This distribution facilitates the stability of the sarcolemma of the muscle fiber during the contraction process.

The DAP complexes can be divided into three groups based on their cellular localization: extracellular (α-dystroglycan and laminin-2), transmembrane (β-dystroglycan, α-, β-, γ-, and δ-sarcoglycan, and sarcospan), and cytoplasmic (dystrophin, α1- and β1-syntrophin, α-dystrobrevin, and neuronal nitric oxide synthase (nNOS); (Figure 1) [9,10]. DAPs link to the F-actin cytoskeleton via dystrophin and to the extracellular matrix via α-dystroglycan. It has been hypothesized that DAPs act as membrane stabilizers during muscle contraction, in order to prevent contraction-induced damage. In addition to their structural roles, they are also thought to mediate cellular signaling in contexts such as mechanical force transduction and cell adhesion [9,10,11].

Pathogenic variants have been reported in the genes of dystrophin (*DMD*), sarcoglycans alpha, gamma, and delta (*SGCB*, *SGCG*, and *SGCD*), and genes of proteins that glycosylate the dystroglycans, such as Protein O-mannosyl transferase 1 and 2 (*POMT1* and *POMT2*), Protein O-mannose beta-1, 2-N-acetylglucosaminyltransferase (*POMGNT1*), Fukutin (*FKTN*), Fukutin-related protein (*FKRP*), and Dystroglycan 1 (*DAG1*). Pathogenic variants in these genes may generate a partial or total absence of DAPs, compromising the stability of the membrane and damaging muscle fibers during contraction [12,13,14]. In patients with MDs, this type of damage causes degeneration of the fiber and, in the long term, death of the muscle structure [14,15]. Among the findings observed in biopsies of individuals affected with pathogenic variants of genes that encode the DAPs, an increase in connective tissue, increase in the number of necrotic fibers, decreased muscle mass, and great variability in the diameter of muscle fibers have been observed. There are also changes associated with fiber regeneration processes, such as internalization of nuclei and bifurcated or poorly formed fibers [16,17,18]. Muscle fiber regeneration processes are associated with the activation of satellite cells, which are mesenchymal stem cells located in the basal lamina of the skeletal muscle [19,20,21]. They mediate the regeneration of muscle tissue through their proliferation, differentiation, and fusion with damaged fibers, contributing to the repair of muscle injury in patients with MDs [22,23,24]. Although satellite cells are still studied in MDs, it is now known that they gradually fail during the progression of the disease, compromising their function and contributing to multiple pathological aspects of the disease [25,26].

At present, there are no treatments for MDs, except for some treatments in very specific cases of DMD with nonsense mutations or the deletion of exon 51, such as Ataluren and Eteplirsen, respectively [27,28]. In an effort to avoid the progressive degeneration of muscle fibers as long as possible, corticosteroid therapies have been used mainly in DMD; however, it seems that there are less therapeutic possibilities for other MDs. This review focuses on current experimental pharmacological treatments that could be affordable in the short term not only for DMD, but for all MDs associated with DAP, until other definitive treatments are established.

## 2. Pharmacological Treatment Applied to Patients with MD

Despite the significant advances in research focused on the understanding of the molecular basis of MDs, in practice, there are only a few advances in treatments applicable to these patients [29,30]. Considering the genetic and phenotypic variability of the different dystrophies described to date, it is a true challenge for modern medicine to standardize an applicable treatment for all MDs. For this reason, most treatments are focused on DMD, as it is the most frequent form of MD worldwide [31]. In this sense, corticosteroids are mainly used in DMD, although some studies have focused on other MDs.

### 2.1. Corticosteroid Treatment in DMD

At present, corticosteroids are part of the care recommendations for DMD and are largely in routine use. This treatment is generally based on the use of prednisone (0.75 mg/kg/day) or deflazacort (0.9 mg/kg/day). Both drugs induce improvements in muscle strength, presentation of scoliosis, respiratory dysfunction, and cardiomyopathy [32,33,34]. Despite their beneficial effects, according to a Cochrane’s systematic review, there is still no sufficient evidence to establish the effect on prolongation of walking—this aspect remains to be elucidated [35]. Treatment should be started between four and six years of age, preferably before the patient loses the ability to walk. Once the patient begins to use a wheelchair, it is recommended to continue or start treatment in order to maintain the function of the upper limbs, reduce the progression of scoliosis and respiratory failure, and to improve cardiac function [31]. The adverse effects that have been reported for this therapy highlight weight gain (mainly with prednisone) and, hence, deflazacort is used more frequently. However, the development of cataracts is greater with deflazacort and, therefore, patients should be monitored by ophthalmologists. The development of osteoporosis due to using both corticosteroids can be reduced through the consumption of vitamin D and calcium [31,36,37]. Interestingly, according to the systematic review mentioned above, a weekend-only prednisone regimen is as effective as daily prednisone in the short term (12 months), with no clear difference in BMI; however, this result was obtained with low-quality evidence [35]. At present, some trials are being conducted to elucidate the long-term effects of a weekend-only prednisone regimen (https://ClinicalTrials.gov accessed on 5 June 2024).

Although a positive effect is clinically observed in patients treated with steroids, there have been few reports on the effects of these treatments at the histological level. In 2015, Peverelli L. et al. analyzed the biopsies of DMD patients not treated with steroids, DMD patients treated with steroids, and healthy controls. They showed that biopsies from one-year-old patients not treated with steroids had more connective tissue (16.50%), compared to those from healthy children (3%), suggesting that the effects on muscle could begin in intrauterine life, although this was not detected clinically. They also determined that during the natural history of the disease, the proportion of connective tissue (CT) remains stable up to six years of age, while between the ages of six and seven, there is a rapid increase in fibrotic tissue. This abrupt increase in the appearance of connective tissue is decreased but still progresses after seven years; in addition, the loss of muscle tissue was concordant with the increase in fibrosis. These findings indicate that the period between six and seven years of age is a crucial time in which muscle tissue loses the ability to self-regenerate, leading to fibrotic degeneration. On the other hand, the authors observed a lower amount of CT in seven-year-old patients treated with steroids (24.82%), compared to steroid-free patients (29.76%). This trend remained observable until 10 years of age, when patients treated with steroids showed 22.38% CT, compared to 30% observed in patients without treatment. The percentage of muscle mass was inversely proportional to the CT, as patients with steroid treatment showed a higher percentage of muscle mass. Other characteristics, such as the proportion of necrotic fibers (tissue necrosis), were increased in patients undergoing treatment, while the percentage of fibers with central nuclei (tissue regeneration) did not present significant differences. This demonstrates that the main effect of corticosteroids consists of decreasing CT (fibrosis) [17]. In experimental models, this is important, as a decrease in fibrosis allows for the proliferation, migration, and differentiation of satellite cells, as well as making myoblast fusion more efficient [19,38,39].

### 2.2. Corticosteroid Treatment in Dystrophies with Alterations in DAPs

Although corticosteroid treatment is part of the clinical guides for patients with DMD, they are not used routinely in any other type of MDs, despite the fact that the progression and histopathology findings of some of them are similar to those of DMD, and that the mechanisms of these disorders are related to destabilization of DAP complexes. For sarcoglycanopathies, different reports of clinical cases of patients with alterations in the *SGCA* (alfa), *SGCB* (beta), and *SGCG* (gamma) genes have shown that patients maintained their muscle strength when corticosteroids were administered [40,41,42,43]. However, in 2014, Albuquerque et al. reported variable responses in the maintenance of muscle strength, as well as pulmonary and cardiological functions, in six patients with sarcoglycanopathy treated with steroids. No clinical improvement was observed, and some patients showed worsening symptoms [44]. As the results remain inconclusive, it is necessary to carry out studies in larger cohorts of patients with a genetic diagnosis to confirm the type of MD, and even to analyze whether specific mutations modify the response to steroids.

Regarding dystroglycanopathies, there are few studies based on clinical reports. In three patients with pathogenic variants of the *FKTN* gene, an important benefit of maintaining independent gait with the administration of 0.75 mg/kg/day of prednisone was reported. When removing steroids, gait deterioration and weakness were observed; hence, the steroids were maintained in these patients [45,46]. According to the above, the use of steroids in MDs associated with *FKRP* could be considered, even in other dystroglcanopaties (i.e., *POMT1*, *POMT2*, *POMGnT1*, and *DAG1*).

## 3. Other Experimental Drugs Used in Models of DAP Alterations

### 3.1. Drug Repurposing

Drug repurposing is the technique of using an existing drug or drug candidate for a new treatment or medical condition for which it was not indicated before. In particular, this provides an avenue for exploring alternative treatment options for MD patients. Pharmacological reuse in DMD is the most explored, where a variety of drugs with beneficial effects on skeletal muscle have been reported. Different investigations in animal models of MDs have reported the effects of various drugs, used for the treatment of bacterial infections (Gentamicin), type II diabetes (Metformin), familial hyperlipidemia (Simvastatin and Pravastatin), breast cancer (Tamoxifen), erectile dysfunction (Tadalafil), gastrointestinal tumors and carcinomas of renal cells (Sunitinib), Parkinson’s disease (safinamide), Leber neuropathy (Idebenone), hypertension (Pargyline and betaxolol), and malignant hyperthermia (Drantolene sodium) [47,48,49,50,51]. These drugs contribute to regulating different pathogenic mechanisms of DMD, such as mitochondrial dysfunction, regulation of ROS, inflammation, and replacement of muscle by connective tissue or depletion of satellite cells, allowing muscle tissue to be maintained under more favorable conditions.

Drug repositioning in other MDs related to DAPs is less studied. Sarcoglycanopathies (mainly *SGCA*), secondary dystroglycanopathies with mutations in the *FKRP* gene, and congenital muscular dystrophy 1A caused by mutation of the laminin alpha-2 gene (*LAMA2*) have been the main targets of drug reuse research. The outcomes observed in animal models with these treatments are complex (Table 1) and, for this reason, exploration of all cellular mechanisms related to the biological effect remains limited. However, the approaches to understand the possible pathways associated with the outcomes show that the regulation of apoptosis, the activation of pathways, such as AKT, regulation of gene expression, regulation of inflammation, and importantly, decreased fibrosis play fundamental roles in the reduction of the dystrophic muscle phenotype, muscle strength gain, increased walking time, and survival in animal models [52,53,54,55,56,57,58,59].

In some studies, relevant clinical doses were shown to be able to translate experimental trials to patients; for example, with Tamoxifen, the authors estimated a human-equivalent dose of 50 mg/day for a weight of 60 kg, a dosing regimen close to that used in breast cancer treatment. From these doses, the side effects observed, such as nausea, dizziness, headache, and depression, may also occur in patients with DAP alterations; however, the risks and benefits must be assessed for each of these drugs.

### 3.2. Novel Molecule Research

The search for new molecules is always of great importance to find cheaper and less toxic alternatives, or those with a better skeletal muscle functional impact in the context of MDs. Various groups have focused on the search for alternatives to entities related to DAPs, with *SGCA*, *FKRP*, and *LAMA2* being the most important targets (Table 2). Although there have been some reports focused on the δ-SG in skeletal muscle, it is important to note that more studies have addressed the dilated cardiomyopathy associated with sarcoglycanopathy, and that molecules such as metformin, epicatechin, and verapamil have been considered to address this problem [60,61,62,63]. Similarly to drug repurposing research, the study of new molecules that may reduce the dystrophic characteristics of the muscle under DAP alterations has been mainly directed at rehabilitating mitochondrial function, inhibition of apoptosis, stability of the sarcolemma, hypertrophy of the fibers, decreased inflammation, and fibrosis [64,65,66,67,68,69].

We considered that the effects of these new molecules and repurposed drugs may possibly have a great impact on the skeletal muscle; however, research so far has been limited to specific points that are of interest to researchers. For this reason, we considered the interaction of different research groups important, as it will allow us to determine the effects of all these molecules on the skeletal muscle at the experimental level in a broader way. Furthermore, it may be shown that the best options can be transferred to clinical studies, which would allow us to determine a real possibility to improve the quality of life of patients with DAP alterations.

## 4. Mechanisms of Experimental Drugs to Regulate Fibrosis in Models with DAP Alterations

### 4.1. Inhibition of Fibrosis

In MDs—mainly those that affect the DAP complexes—there is a fragile sarcolemma generating a chronic state of damage to the muscular fiber. Therefore, the highly controlled processes of regeneration become imbalanced. Remodeling of the extracellular matrix (ECM), which is a normal process within muscle repair, is deregulated in MDs, accumulating components in the ECM—mainly collagens—that replace damaged muscle fibers and generate fibrotic and scar tissue [70,71,72]. Of note, most repurposed molecules regulate the activity of fibroblasts, such as Nintedanib, Rapamycin, Tamoxifen/raloxifene, N-acetyl-L-cysteine (NAC)/Vitamin E, and Losartan [73,74,75,76,77,78,79,80]. Regarding studies of novel molecules, the regulation of fibroblasts has only been reported for Halofuginone and Omigapil (Table 1 and Table 2) [81,82,83]. With the exception of Nitroflurbiprofen (HCT1026) and Thiostrepton, most of the molecules analyzed in MDs with alterations in DAPs have been studied in fibroblast models (Table 3). This suggests that the effects of these molecules have an important role in regulating the formation of fibrosis and scar tissue, which merits further investigation. The regulation of the activity of fibroblasts by these molecules is mediated through different mechanisms.

#### 4.1.1. The TGF-β/Smad Pathway

**Figure 2:**
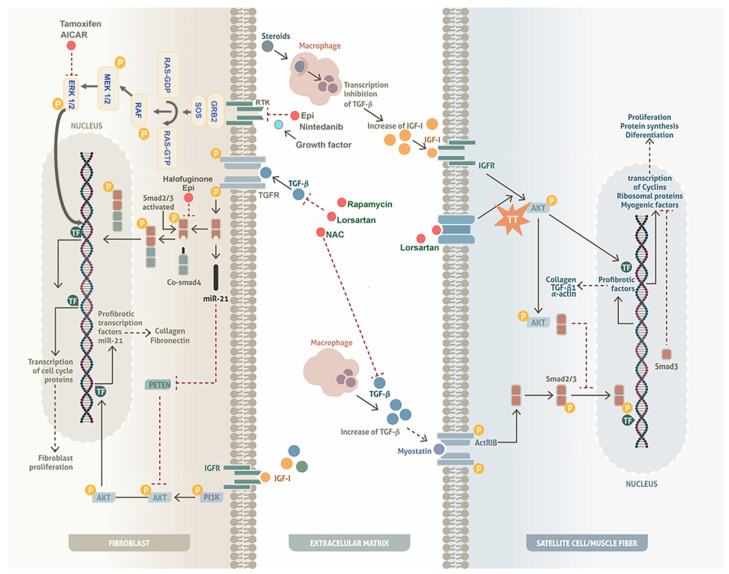
Effects of drugs on fibroblasts, satellite cells, and muscle fibers. The function of TGF-β (stored in the extracellular matrix or produced by skeletal muscle macrophages) is inhibited by different drugs, which prevents the binding of TGF-β to its receptor on fibroblasts. Inhibiting the Smad2/3 pathway prevents the transcription of profibrotic genes. Other drugs can inhibit fibroblast proliferation through negatively regulating mir21/PTEN/AKT or ERK1/2. In muscle fibers, TGF-β activation contributes to the interaction of myostatin with its receptor, initiating the Smad2/3 pathway and favoring the expression of profibrotic factors. The administration of corticosteroids decreases the expression of TGF-β and increases the IGF-1/AKT/mTOR pathway in muscle fibers, favoring protein synthesis. Other drugs, such as Losartan, through binding to the AT1 receptors of muscle fibers, enhance the activation of the AKT pathway for protein synthesis and the inhibition of profibrotic factors due to the interaction of AKT with Smad2/3. In satellite cells, AKT activation contributes to promoting proliferation and inhibits the function of Smad3, allowing the correct functioning of myogenic factors, such as MyoD, and thus contributing to the differentiation process. P: Phosphorylated proteins.

Transforming growth factor beta (TGF-β) is known to participate in various cellular processes, including differentiation, proliferation, migration, extracellular matrix (ECM) remodeling, and apoptosis, all of which influence inflammation and fibrosis [87,88]. TGF-β is stored in the ECM and is activated by tissue damage or specific growth signals [89]. In different experimental animal models, losartan, NAC, Rapamicin, Halofuginine, and (−)-Epicatechin have been shown to have effects on regulation of the (TGF-β)/Smad pathway in fibroblasts (Table 3) [86]. These molecules inhibit the binding of TGF-β to its receptor in the fibroblasts of the muscle’s ECM, preventing the canonical Smad 2/3 pathway from being activated with co-Smad4. Consequently, these transcription factors cannot be translocated to the nucleus, which inhibits the transcription of profibrotic genes that generate the synthesis of ECM proteins, such as collagen and fibronectin (Figure 2) [90]. The effect of TGF-β on the regulation of Smad2 is also related to the function of miR-21 in fibroblasts, which generates a persistent activation of the AKT pathway by inhibiting PTEN. This AKT/PTEN deregulation generates a hyperproliferative phenomenon and cell survival in fibroblasts (Figure 2) [91,92]. Furthermore, these molecules could contribute to inhibition of fibroblast proliferation through regulating the function of miR-21/Smad in dystrophic models. It has been shown that losartan contributes significantly to the reduction in fibrosis formation through regulation of the TGF-β/Smad pathway and through regulation of miR-21 in dystrophic models [93]. Nevertheless, more studies are needed in this context.

On the other hand, muscle fibers and satellite cells may also respond to those molecules. In a disuse atrophy model, losartan administration blocked the angiotensin II type I (AT1) receptor in muscle fibers through increasing activation of the IGF-1/AKT/mTOR pathway [94]. Positive activation of mTOR increases protein synthesis and inhibits protein breakdown, preventing muscle loss (Figure 2) [95]. In other studies, it has been observed that activation of the AKT pathway also has an impact on the regulation of fibrosis. Moreover, it has been shown that AKT in satellite cells and muscle fibers binds with Smad3 (AKT/Smad3), preventing translocation of Smad3 to the nucleus and inhibiting the expression of genes, such as TGFβ1, collagen I, and α-actin (Figure 2) [93]. It has also been reported that TGF-β, through the Smad3 pathway, represses the transcriptional activity of myogenic differentiation 1 (MyoD), which inhibits muscle differentiation [94]. Therefore, inhibition of the TGF-B/Smad pathway not only plays a fibroblast regulatory role, but also favors a better response of satellite cells and myofibers. Hence, MDs due to alterations in DAPs could benefit from administration of losartan. In experimental models, losartan played an important role in regulation of fibrosis and inhibition of muscle mass loss, possibly through the regulation of Smad3, as it contributes to the adequate differentiation of satellite cells.

#### 4.1.2. Extracellular Signal-Regulated Kinase (ERK) Pathway

The MAPK cascades are intracellular signal transduction pathways that regulate a wide variety of cellular processes, including proliferation, differentiation, apoptosis, and stress responses [96]. Dysregulation or improper functioning of MAPK cascades is involved in the induction and progression of diseases. The ERK1/2 cascade is considered as a prototype of these kinase cascades, which plays a central role in the signaling of a wide variety of extracellular agents that operate via various receptors [96,97]. ERK is activated by several extracellular stimuli, such as growth factors, cytokines, hormones, and heat or oxidation stress through receptor tyrosine kinases (RTKs) or EGFR [96,97,98,99].

This receptor–ligand recognition activates the RAS-related GTPases, which acts as a switch in growth signaling. RAS changes from the inactive GDP-bound form to the active form, then undergoes a conformational change, leading to the binding and regulation of RAF, which phosphorylates and activates the serine/threonine-protein kinases MEK1/2. These, in turn, phosphorylate and activate ERK1/2 kinases. Activated ERK1/2 kinases phosphorylate a number of substrates, including cytosolic signaling proteins, transcription factors that regulate the expression of genes involved in proliferation, differentiation, and cell cycle progression. Nuclear translocation of ERK leads to cell growth through increased expression of cyclin D and subsequent progression from the G1 to the S phase of the cell cycle [100,101,102]. The ERK 1/2 pathway, activated by receptor tyrosine synase (RTK), is blocked by Nintedanib and Halofuginone; the latter is interesting, as it has been shown to possess the ability to inhibit both pathways related to the activation of fibroblasts (Figure 2) [73,74,83,103]. The drugs AICAR and Tamoxifen also have an effect on this pathway, although the mechanism has not yet been clarified; however, the inhibition of ERK 1/2 is evident, preventing translocation to the nucleus and inhibiting the ability to proliferate and generate a chronic fibrotic state [76,84,85]. Considering the above, it is necessary to elucidate the beneficial effects observed in murine models with DAP alterations.

## 5. Antioxidants as Possible Pharmacological Molecules in MDs with Alterations in DAPs

Although oxidative stress normally contributes to muscle fiber homeostasis, its increase in MDs has an important implication for the development of weakness [104]. Oxidative stress promotes the invasion of inflammatory cells, interferes with cell signaling that promote muscle repair, impairs mitochondrial function, and increases necrosis and apoptosis [105]. Disruption of the DAP complexes increases oxidative stress due to the loss of neuronal nitric oxide synthase (nNOS), which also binds to the complexes. The loss of nNOS affects the action of nitric oxide (NO), which is a messenger molecule that transduces signaling events in a calcium-dependent manner and is capable of regulating muscle development, contraction, and blood flow [106]. Loss of nNOS also increases cell susceptibility to superoxides through increasing the activity of NAD(P)H [107,108]. This oxidative stress increase can produce superoxide anions and hydrogen peroxide. In turn, these molecules are converted to reactive oxygen species (ROS) and reactive nitrogen species (RNS). ROS and RNS are highly reactive with the membrane lipids and affect the structural proteins and DNA of muscle fibers [109,110].

**Figure 3:**
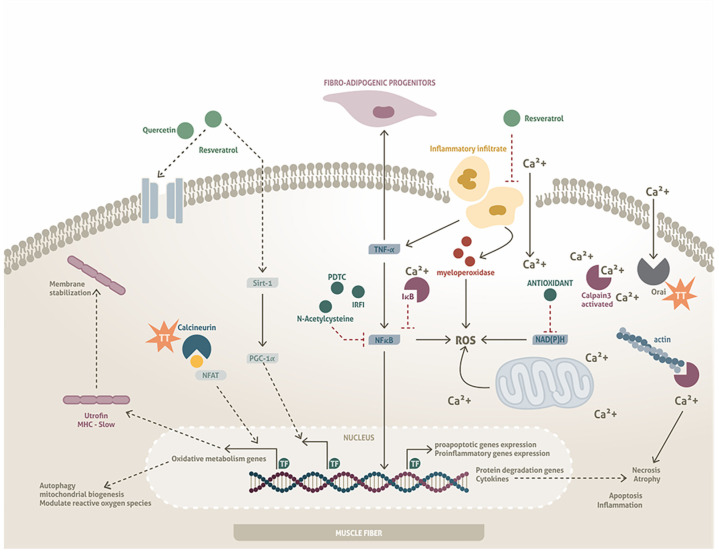
Effects of antioxidants and possible therapeutic targets in dystrophic muscle. Antioxidants (PDTC, IRFI, and N-acetylcysteine) inhibit NF-kB, repressing the expression of genes associated with apoptosis, inflammation, and atrophy; furthermore, the activation of fibro-adipogenic progenitors is also decreased. Resveratrol induces oxidative metabolism regulation through the regulation of Sirt-1 and ROS through NAD(P)H and reduces the inflammatory infiltrate via mechanisms that are not yet clear. Inhibition of the Orai protein can decrease the necrosis and fibrosis observed in muscular dystrophy tissues, with the main pathway being calpain-3 downregulation and NF-κB inhibition. On the other hand, the inhibition of Orai increases NFAT expression, which contributes to the elevation of proteins, such as utrophin and MHC-I, which provide greater stability to the membrane and generate a replacement of the fiber type, respectively.

In mdx mice treated with antioxidants, increased fiber regeneration and reduced fatigue were observed, ameliorating the dystrophic phenotype [111]. Various antioxidants, such as pyrrolidine dithiocarbamate (PDTC), N-acetylcysteine, IRFI-vitamin E, and diapocynin, have been shown to play an important role in inhibiting the function of NF-κB (Figure 3) [111,112,113,114]. NF-κB is a transcription factor with multiple levels of regulation that mediates various biological functions, such as the expression of genes related to the ECM, protein degradation, inflammation, and regulation of the immune system [115,116,117]. Chronic activation of the NF-κB pathway is considered to play an important role in skeletal muscle degeneration in MD patients, as it maintains macrophage activation, promoting inflammation and necrosis of muscle fibers and limiting their regeneration through inhibition of satellite cell differentiation [117].

Other antioxidants, such as resveratrol and quercetin, exert their activity through another mechanism of action characterized by activation of the protein/histone deacetylase Sirt1, which is an important regulator of cytoplasmic and nuclear proteins, such as p53, FOXO, HIF-1α, and peroxisome activation receptor-1 coactivator (PGC-1α) [65,66,67]. PGC-1α is a transcription factor that plays an essential role in regulating genes involved in balancing oxidative metabolism, thus modulating the production of ROS, autophagy, and mitochondrial biogenesis in muscle [107]. Furthermore, Sirt1 activation has also been associated with increased expression of utrophin, a homologue of the protein dystrophin (Figure 3) [118,119]. Utrophin is located primarily at the neuromuscular junction but can also be expressed in the sarcolemma. Utrophin partially contributes to restoring the bridge between the ECM and the cytoskeleton in animal models which, importantly, reduces the phenotype of the pathology [120].

Gordon et al. reported that resveratrol at a dose of 100 mg/kg reduced the infiltration of neutrophils and macrophages, in addition to increasing the expression of PGC-1α [121]. Although the utrophin transcript was elevated in this study, protein expression in the muscle was not different in resveratrol-treated mice. However, utrophin, PGC-1α, and IL-6 have been found to be elevated during muscle differentiation; therefore, it is possible that they contribute to the differentiation process, although these processes still need to be clarified (Figure 3). The decrease in inflammatory infiltrate invasion with resveratrol is interesting as, in addition to reducing oxidation, it can also decrease the expression of TNF-α, a cytokine that is capable of activating fibro-adipogenic progenitors [122,123]. Therefore, the contributions of antioxidants to the inhibition of fibrosis also deserve further study.

## 6. Evaluation of Target Sites as New Therapeutic Strategies

### 6.1. Calcium Regulation

The abnormal Ca^2+^ homeostasis observed in patients with MDs suggests that this ion plays an important role in the development of the disease. One of the main mechanisms by which elevated Ca^2+^ causes muscle wasting is due to the activation of calcium-dependent proteases, such as calpain 3, a calcium-dependent cysteine protease expressed in skeletal muscle, whose deficiency has been associated with Limb-girdle muscular dystrophy type 2A (LGMD2A). Calpain 3, activated by an intracellular increase in Ca^2+^, degrades the IkBα subunit of the NF-kB complex, thus activating its transcriptional capacity, which contributes to the expression of pro-apoptotic, pro-inflammatory, and protein degradation genes in the muscle fiber. Calpain 3 also stimulates the release of cytokines that prevent muscle regeneration and, eventually, promotes the degradation of specific proteins of the cytoskeleton and sarcoplasmic reticulum, leading to the characteristic necrosis of muscle tissue (Figure 3) [124].

Studies in mdx mice have reported a significant elevation of Ca^2+^ in the cytoplasm, but it has already been shown that this elevation is not uniform in the cytosol. This phenomenon is restricted to the subsarcolemmal space, which seems to indicate that the main entry of Ca^2+^ is due to injury to the muscle fiber membrane [125,126]. However, in order to understand whether only the microfracture of the sarcolemma causes the Ca^2+^ dysregulation observed in dystrophies, a mouse model has been developed. A transgenic mouse over-expressing transient receptor potential canonical 3 (TRPC3)—a family of non-selective cation channels over-expressed in patients with MD—presented an increase in Ca^2+^ influx resulting in a severe dystrophic pattern, with a marked fibrosis process, presence of fatty tissue, degeneration, and infiltration of immune cells. These results suggest that MD may also be initiated by the deregulation of calcium-dependent mechanisms [127]. Ca^2+^ regulation, secondary to membrane injury or primary in the development of muscle disorders, is a target of interest to modify the dystrophic phenotype.

Capacitive or store-operated calcium entry is a pathway for Ca^2+^ entry when intracellular stores are reduced [128,129]. This pathway depends on two proteins: stromal interaction molecule 1 (STIM1) and calcium-release-activated calcium modulator (Orai). STIM1 functions as a sarcoplasmic reticulum Ca^2+^ sensor, translocating from the reticulum membrane to nearby regions of the plasma membrane when cytoplasmic Ca^2+^ concentrations have decreased. This movement of STIM1 activates the Orai protein, which is a pore-forming unit that allows Ca^2+^ to enter the cytosol through the plasma membrane [130,131,132]. In dystrophic mice, the expression of Orai was upregulated, unlike STIM1; therefore, it can be considered that Orai may play an important role in Ca^2+^ regulation in dystrophic models. Blockade of the STIM1/Orai pathway reduces calpain activity in dystrophic fibers (Figure 3) [133]; however, it is necessary to study whether there are histological and physiological changes that contribute to the preservation of muscle structure and function.

On the other hand, Ca^2+^ homeostasis in the cytoplasm of the muscle fiber plays an important role, as it directly regulates gene expression through calcium-dependent pathways, such as calmodulin/NFAT. In response to increased Ca^2+^, NFAT is dephosphorylated by calmodulin-dependent calcineurin phosphatase, resulting in its translocation to the nucleus and subsequent contribution to the expression of genes which, in the particular case of muscle fibers, include Myosin heavy-chain-1 (MHC-I), myoferlin, utrophin A, and Myocyte-specific enhancer factor 2C (MEF2C) [134,135,136]. In dystrophic muscles, Ca^2+^ dysregulation can activate pathways that antagonize the calcineurin/NFAT pathway, resulting in the expression of necrosis and apoptosis genes, thus contributing to progression of the pathology. Interestingly, transgenic expression of calcineurin in dystrophic muscle resulted in a nuclear increase in NFATc1, generating a change in fiber phenotype (slow phenotype), increasing utrophin levels, and providing integrity to the sarcolemma (Figure 3) [137,138]. Therefore, it is clear that Ca^2+^ regulation is one of the most important targets for reduction of the dystrophic phenotype and, although there are more possibilities to influence Ca^2+^ regulation, we propose that Orai, TRPC3, and NFAT may be relevant therapeutic targets.

### 6.2. Regulation of Hypertrophy/Proliferation/Differentiation

In mouse models of DMD/BMD (mdx/utr^-/-^) and LGMD, and a null model for the sarcoglycan delta gene (sgcd^-/-^), the development of hypertrophy is a common process in the course of the disease. As such, it has been proposed that this is a compensatory mechanism in response to fiber degeneration [20]. A molecular mechanism that has been clarified is an increase in the activation of phosphorylated serine/threonine kinase (pAKT), whose function is to activate the downstream mTORC1/p70S6K complex, which is responsible for protein synthesis. The enhanced activation of pAKT occurs as part of the regenerative response linked to muscle hypertrophy during the pre-necrotic stages of the disease and may contribute to reducing the severity of the pathology [139,140].

The Akt-transgenic mdx mice presented conditional activation of AKT along with doxycycline. This model showed changes in the AKT/mTOR/p70S6K pathway, when compared to mdx mice with normal pAKT activity. Activation of the Akt transgene at the pre-necrotic stage of the disease (<3.5 weeks of age) delayed the onset of dystrophic pathology, increased muscle hypertrophy, and decreased sarcolemmal fragility. This is due to the over-expression of utrophin, which compensated for the loss of DAPs in Akt-transgenic mdx mice (Figure 3) [16]. These findings indicate the AKT molecule as a possible therapeutic target to reduce the dystrophic phenotype in early stages of the disease. In addition, through different interventions in animal models and in cell cultures, other authors have also shown that the mechanism that reduces the dystrophic pathology is related to AKT activation [141,142,143,144]. These results indicate the AKT molecule as a possible therapeutic target for MD. Therefore, it is essential to search for molecules that can activate this pathway to determine whether they can help to reduce the dystrophic phenotype.

As the muscle fibers cannot divide after injury, repair relies on satellite cells; therefore, AKT activation may contribute to satellite cell division (Figure 2). In addition to its effect on proliferation, AKT can contribute to the expression of transcription factors that promote myoblast differentiation, intervening in the expression of myogenic factors, such as MyoD (Figure 2). Both processes, proliferation and differentiation, play important roles in muscle repair and the development of hypertrophy.

## 7. Conclusions

Corticosteroids are the current treatment for DMD—the most frequent MD. While their use has demonstrated beneficial effects in DMD, their effects are still not clear in the context of other MDs associated with DAP disruption. Until a definitive treatment for MDs is obtained, it is necessary to develop studies in order to elucidate whether corticosteroids or other drugs (e.g., repurposed drugs or antioxidants) could benefit MD patients. Undoubtedly, the regulation of fibrosis, inflammation, and mitochondrial function is crucial in these conditions. Nevertheless, other processes, such as the regulation of Ca^2+^ and pathways associated with protein catabolism observed in different types of MDs, can serve as therapeutic targets, even at early stages of the disease. Drug repurposing for MDs is a promising field. Different investigations in animal models have demonstrated that some drugs may regulate different pathogenic mechanisms, mainly in DMD, but also in some other MDs. Novel molecules (e.g., v.gr., HCT1026, and nitric oxide) can inhibit fibrosis or inflammation, among other effects, thus having a great impact on the improvement of skeletal muscle damage. Oxidative stress has important implications in the development of weakness in MD, as it can interfere with muscle repair and promote necrosis, apoptosis, and inflammation. Murine models have shown that antioxidants can repress these effects. Abnormal Ca^2+^ homeostasis is characteristic of MD, and some Ca^2+^ regulators (Orai, TRPC3, and NFAT) can be considered as therapeutic targets for MDs.

Several drugs and molecules explored in this review have shown beneficial effects; nevertheless, the great majority of the studies have been carried out using cellular or animal models. Therefore, the next step is translational research; that is, the direct application of these scientific discoveries in clinical practice. Once their effects are better defined, it will be necessary to carry out clinical trials to analyze their effects in the “real world”.

## Figures and Tables

**Table 1:** Effects of repurposing drugs in muscle models with alterations in DAPs.

Disease	Protein	Dose (Time Treatment)	Original Indication	Study Model	Outcomes	Pathways Modified	Reference
Sarcoglycanopathy	α-SG	Bortezomib and Givinostat (5 nM and 10 uM/ 24 h)	Multiple myeloma and DMD	R77C-α-SGmCh (independent triplicate)	R77C-α-SG membrane expression rescue	Blocks autophagy and proteasome	[52]
		Nintedanib (50 mg/kg twice daily/10 weeks)	Pulmonary fibrosis	*Sgca*-/- mice (n = 21 ^†,¥^)	Results in a functional muscle improvement, attenuating muscle fibrosis and inflammation	Reduction in the expression of some genes that are also involved in inflammation	[53]
Secondary Dystroglycanopathies	FKRP	Rapamycin (2 mg/kg once a day/4 weeks)	Immunosuppression	Myf5/Fktn conditional KO (n = 12 ^¥^)	Reduction of fibrosis and muscle damage improves muscle function	Partial regulation of autophagy and increased mTORC1 activation	[54]
		Tamoxifen and raloxifene (50 mg/kg and 100 mg/kg/1 year)	Breast cancer and osteoporosis	P448L mice (n = 10 ^†^)	Reduces muscle degeneration, reduces fibrosis, improves muscle functions, respiratory functions, and bone density	Inhibition of TGF-β and PAI-1, inhibition of fibroblast proliferation, inhibition of protein kinase C and NF-kB pathway	[55]
Congenital muscular dystrophy type 1A (MDC1A)	Laminin-α2	N-acetyl-L-cysteine (NAC) and Vitamin E (150 mg/kg and 40 mg/kg, six times a week for 22 and 14 days, respectively)	Acetaminophen overdose, cystic fibrosis, and antioxidant activity	Homozygous dy2J/dy2J mice (n = 5 ^§^)	Preserves muscle strength, reduces central nuclei, apoptosis, inflammation, fibrosis, and oxidative stress	Inhibits the upregulation of Fn1 gene expression in a differential manner in muscles	[56]
		Doxycycline (6 mg/mL 6 weeks)	Antibiotic	Heterozygous Lama2^dy-W/+^ (n = 9)	Increases the median lifespan, increases body weight, delays hindlimb paralysis, reduces inflammation, inhibits apoptosis, delays the appearance of functional defects in motor nerves	Increases Akt phosphorylation, inactivation of Bax, and decreases caspase-3 activity	[57]
		Losartan 0.6 g/l, 12 weeks	Hypertension	Homozygous dy^2J^/dy^2J^ (n = 12 ^†^)	Promotes survival, improves muscle strength, reduces fibrosis	Inhibits TGF-β and MAPK, increases TNF-α and mRNA expression of TRAF1, TRAF2, CIAP2, and FTH-1, increases BCL2, decreases caspase-3	[58,59]

^†^ Groups that include females and males. ^¥^ The WT group had a smaller number of animals. ^§^ The WT group had a greater number of animals.

**Table 2:** Effects of novel molecules in muscle models with DAP alterations.

Disease	Protein	Drug Name	Dose (Treatment Time)	Study Model	Skeletal Muscle Effect	Reference
Sarcoglycanopathy	α-SG	HCT 1026 and nitric oxide	30 mg/kg (for up to 12 months)	α-SG-null mice (n = 9)	Inhibition of inflammation. Preservation of satellite cell number and activity.	[64]
		Thiostrepton	3 μM (24 h)	R77C-α-SGmCh (independent triplicate)	Rescue of different mutations in α-SG. Membrane rescue.	[65]
	δ-SG	(−)-Epicatechin	1 mg/kg (twice a day for 2 weeks)	B6.129-Sgcdtm1Mcn/J mice (n = 5)	Recovery of reduced/ oxidized glutathione. Enhanced superoxide dismutase 2, catalase, and citrate synthase activities.	[66]
Secondary Dystroglycanopathies	FKRP	AICAR	500 mg/kg (4 weeks)	Myf5/Fktn knockout (n = 7 ^†,§^)	Recovery of muscle contractile function. Enhancing autophagy. Induces satellite cell proliferation.	[69]
Congenital muscular dystrophy type 1A (MDC1A)	Laminin-α2	Halofuginone	5 μg (three times a week for 5 or 15 weeks)	dy2J/dy2J mice (n = 5)	Inhibited muscle fibrosis. Reduced collagen levels. Reduced number of centrally nuclear myofibers. Increased myofiber diameter.	[68]
		Omigapil	0.1 or 1 mg/kg	dyw/dyw mice (n = 13 ^¥^)	Reduces apoptosis and fibrotic tissue.	[67]

^†^ Groups that include females and males. ^¥^ The control/WT group had a smaller number of animals. ^§^ The control/WT group had a greater number of animals.

**Table 3:** Effects of experimental drugs used in models of muscular dystrophies associated with DAPs on fibroblasts.

Drug/Molecule	Experimental Model	Mechanism of Action/Effect	Reference
AICAR	NRK-49F rat renal fibroblast cell line	Partially mediated through the activation of AMPKα-1.	[84,85]
		Downregulation of ERK 1/2.	
	Mesenchymal fibroblasts derived from Hearts rats	Mesenchymal fibroblasts derived from Hearts rats.	
NAC	Hepatic stellate cells isolated from male rats	Interferes with TGF-β.	
	Sprague Dawley rats	The phosphorylation of receptor Smads by TβRI is abolished.	[77,78]
		Decrease in ligand affinity of the type III receptor (TβRIII) complex.	
	Human fetal lung fibroblast (HFL-1) cells	Inhibits the increase in TGF-β1.	
		Inactivates TGF-β1 itself and inhibits binding to its receptor.	
Rapamycin and	Lung primary fibroblasts	Inhibits ECM protein expression.	
Pirfenidone		Inhibits the synthesis of profibrotic markers induced by TGF-β.	[75]
Tamoxifen	Primary human skin fibroblasts (AG1523)	Inhibits ERK1/2 signaling.	
	Human breast fibroblasts (HBF1 and HBF12)	Inhibits the activation of fibroblasts.	[76]
Nintedanib	Biopsies from patients with systemic sclerosis (SSc)	Reduces TGF-β-induced fibrosis.	[73,74]
		Reduced proliferation and migration of fibroblasts, as well as myofibroblast differentiation and collagen release.	
	Primary human lung fibroblasts	Antiproliferative capacity.	
		Receptor tyrosine kinase inhibitor.	
(−)-Epicatechin	Cardiac fibroblasts	Decreases TGF-β1 levels.	[86]
		Decreases fibronectin, urea, proline, and total collagen protein levels.	
		Restores levels of estrogen receptor (GPER).	
		Effects on the SMAD/TGF-β1 pathway, decreases SMAD levels.	
Halofuginone	Tight skin mouse (TSK) model for scleroderma	Blocked the phosphorylation and subsequent activation of Smad3 downstream of TGFβ signaling.	[82,83]
	Fibroblasts isolated from rat renal papillae	Inhibition of proliferation.	
		Inhibition of PDGF.	
Bortezomib	Adult human lung fibroblast lines	Inhibits FGF-2-induced fibroblast proliferation.	
	(CCL-210 and MRC5)	Prevents TGF-β-induced fibroblast differentiation.	
		Inhibits key kinases activated by TGF-b and FGF-2.	[80]
Losartan	C57BL/6 J mice, unilateral ureteral obstruction (UUO)	Degradation of TβRI through expression of Smurf1 and Smurf2.	
	Spontaneously hypertensive rats (SHRs)	Inhibition of the classical TGF-β/Smad pathway.	[79]

## Data Availability

Data are available from the corresponding authors upon reasonable request.

## Abbreviations

Smad 2/3	Mothers against decapentaplegic homolog 2/3
Smad 4	Mothers against decapentaplegic homolog 4
miR-21	microRNA-21
PTEN	Phosphatase and tensin homolog
AKT	Serine/threonine kinase
IGF-1	Insulin-like growth factor 1
mTOR	Mammalian target of rapamycin
NADPH	Nicotinamide adenine dinucleotide phosphate
FOXO	Forkhead box O
HIF-1	Hypoxia-inducible factor 1-alpha
NFAT	Nuclear factor of activated T-cells
p70S6K	Ribosomal protein S6 kinase beta-1
